# The Complexity of Blood Pressure Fluctuation Mediated the Effects of Hypertension on Walking Speed in Older Adults

**DOI:** 10.3389/fnagi.2021.640942

**Published:** 2021-04-29

**Authors:** Xin Jiang, Yurun Cai, Yue Zhao, Xia Gao, Dan Peng, Hui Zhang, Wuhong Deng, Wen Fu, Na Qin, Ruizhen Chang, Brad Manor, Junhong Zhou

**Affiliations:** ^1^Department of Geriatrics, Shenzhen People’s Hospital, Shenzhen, China; ^2^The Second Clinical Medical College, Jinan University, Shenzhen, China; ^3^The First Affiliated Hospital, Southern University of Science and Technology, Shenzhen, China; ^4^Department of Epidemiology, Johns Hopkins Bloomberg School of Public Health, Baltimore, MD, United States; ^5^Department of Electrical and Computer Engineering, University of Rochester, Rochester, NJ, United States; ^6^Department of Neurology, Shenzhen People’s Hospital, Shenzhen, China; ^7^Hinda and Arthur Marcus Institute for Aging Research, Hebrew SeniorLife, Roslindale, MA, United States; ^8^Division of Gerontology, Beth Israel Deaconess Medical Center, Boston, MA, United States; ^9^Harvard Medical School, Boston, MA, United States

**Keywords:** hypertension, walking speed, older adults, white matter hyper intensities, blood pressure fluctuation, multiscale entropy

## Abstract

**Background:** Older adults with hypertension often had diminished walking performance. The underlying mechanism through which hypertension affects walking performance, however, has not been fully understood. We here measured the complexity of the continuous systolic (SBP) and diastolic (DBP) blood pressure fluctuation, grade of white matter lesions (WMLs), and cognitive function and used structural equation modeling (SEM) to examine the interrelationships between hypertension, BP complexity, WMLs, cognitive function, and walking speed in single- and dual-task conditions.

**Methods:** A total of 152 older adults with age > 60 years (90 hypertensive and 62 normotensive participants) completed one MRI scan of brain structure, a finger BP assessment of at least 10 min, Mini-Mental State Examination (MMSE) to assess cognitive function, and 10-meter walking tests in single (i.e., normal walking) and dual tasks (i.e., walking while performing a serial subtraction of three from a random three-digit number). The grade of WMLs was assessed using the total score of Fazekas scale; the complexity of SBP and DBP was measured using multiscale entropy (MSE), and the walking performance was assessed by walking speed in single- and dual-task conditions.

**Results:** As compared to normotensives, hypertensive older adults had significantly slower walking speed, lower complexity of SBP and DBP, greater grade of WMLs, and poorer cognitive function (*p* < 0.03). Those with lower BP complexity (β > 0.31, *p* < 0.003), greater WML grade (β < −0.39, *p* < 0.0002), and/or poorer cognitive function (β < −0.39, *p* < 0.0001) had slower walking speed in single- and/or dual-task conditions. The SEM model demonstrated significant total effects of hypertension on walking speed, and such effects were mediated by BP complexity only, or BP complexity, WML grade, and cognitive function together.

**Conclusion:** This study demonstrates the cross-sectional association between the complexity of continuous beat-to-beat BP fluctuation, WML grade, cognitive function, and walking speed in hypertensive and normotensive older adults, revealing a potential mechanism that hypertension may affect walking performance in older adults through diminished BP complexity, increased WML grade, and decreased cognitive function, and BP complexity is an important factor for such effects. Future longitudinal studies are warranted to confirm the findings in this study.

## Introduction

Walking safely is the fundamental of everyday activities. The age-associated decline in the performance of walking (e.g., slowed walking speed) is highly prevalent in older adults and has been associated with diminished quality of life (Blacklock et al., 2007), increased risk of falls (Wajda et al., 2013), and cognitive impairment (Montero-Odasso et al., 2017). As we know, aging has become a global issue, and the proportion of older adult population has exceeded 7% globally (Chen et al., 2019). In many countries, the aged society has emerged. In China, for example, the percentage of older adults has reached 12% in 2020 (Chen et al., 2019). Therefore, it is highly demanded to advance our understanding of the age-related decline in walking and the factors associated with slowed walking speed in older adults, which may help develop better rehabilitative strategies to reduce the fall risk for older adults who have problems in walking.

Hypertension is a highly prevalent vascular condition in older adults and an independent risk factor for the problems of walking (Dumurgier et al., 2010; Hajjar et al., 2011; Rosano et al., 2011). Studies have shown that, for example, compared to normotensive older adults, the hypertensive older adults walked significantly more slowly (Dumurgier et al., 2010) and had greater annual decline in walking speed in concurrent with the cognitive impairment and mood problems (Hajjar et al., 2011; Rosano et al., 2011). However, *the underlying mechanisms through which hypertension affects walking performance in older adults are not fully understood*. One potential mechanism underlying the diminished walking performance in hypertension is the pathological change in vascular systems. The long-term exposure to abnormal blood pressure (BP) control in hypertension alters the regulation in both cardiovascular and cerebral vascular systems, leading to vascular impairments [e.g., white matter lesions (WMLs)] (Chou et al., 2018). The white matter contains numerous nerve fibers and axons that connect the cerebral regions of the brain. The WMLs disrupt these important connections and communications, resulting in altered functions, such as the impaired cognitive function pertaining to diminished walking performance in older adults (Srikanth et al., 2009; Aronow, 2017). It is thus of great significance to explore the interrelationship between the characteristics of BP fluctuation, the grade of WMLs, its related cognitive impairment, and diminished walking performance.

The BP is regulated by numerous bio-physiologic procedures and their interactions over different temporal scales, such as the neural feedback happening at milliseconds and the circadian rhythmic changes over hours or days (Douma and Gumz, 2018). Therefore, the dynamics of the continuous beat-to-beat BP fluctuation are “complex,” and these complex BP patterns over multiple scales contain rich physiologically meaningful information. The degree of such physiologic complexity in BP can be captured using techniques derived from complex system theory, including multiscale entropy (MSE) (Costa et al., 2002), a well-developed and widely used technique that quantifies the entropy or recurrence in physiologic series over different temporal or spatial scales. Studies have shown that the complexity metrics can capture the level of the functionality of physiologic systems (i.e., the less complex the physiologic fluctuation, the poorer the system’s functionality (Manor and Lipsitz, 2013)), while traditional metrics measuring the characteristics on a single scale, such as the mean or standard deviation, cannot (Zhou et al., 2016, 2017; Rangasamy et al., 2020). Rangasamy et al. (2020), for example, used MSE to measure the complexity of the beat-to-beat BP fluctuation and demonstrated that lower preoperative BP complexity was associated with a higher estimated risk of adverse cardiovascular outcomes during surgery, while no association was observed between the mean level or variability of BP and the risk. Moreover, we recently observed in a prospective study that lower BP complexity at baseline in older adults was associated with the risk of dementia or Alzheimer’s disease in the next 19 years (Ma et al., 2020). These findings indicate that the multiscale dynamics of continuous BP fluctuation, as measured by complexity, would be a novel marker capturing the subtle pathological changes in vascular systems. However, the mechanism through which this characteristic is associated with diminished walking performance is still unknown. We here thus contend that the alterations of BP regulation in hypertension may manifest in the loss of BP complexity, and lower BP complexity would contribute to the effects of hypertension on slower walking speed in older adults.

To test the overall hypothesis, in this study, we measured the complexity of the beat-to-beat BP series and assessed the grade of WMLs, cognitive function, and walking speed in single- and dual-task (i.e., walking while performing a concurrent cognitive task) conditions in a group of older adults. We then implemented a structural equation modeling (SEM) method to assess the interrelationship between hypertension, BP complexity, WML grade, cognitive function, and walking speed. Specifically, we hypothesize that (1) compared to the normotensive cohort, hypertensive older adults would have lower BP complexity, greater WMLs, poorer cognitive function, and slower single- and dual-task walking speed; (2) lower BP complexity, greater WMLs, and/or poorer cognitive function would be associated with slower walking speeds; and (3) the relationship between hypertension and walking speeds may be mediated by BP complexity, WMLs, and cognitive function.

## Materials and Methods

### Participants

We searched in a data repository consisting of 200 older adults who had clinical visits in recent 12 months in the Department of Geriatrics, Shenzhen People’s Hospital, and expressed interests in future studies. Three study staffs then contacted them and completed the screening on those who can participate in this study. The inclusion criteria for both hypertensive and normotensive cohorts were (1) age greater than 60 years and (2) with the ability to walk for at least 1 min without physical assistance. The inclusion criterion for the hypertensive cohort was clinically diagnosed hypertension (i.e., SBP ≥ 140 mmHg or DBP ≥ 80 mmHg) by measuring the brachial artery of the right arm using a sphygmomanometer, and that for the normotensive cohort was SBP < 140 mmHg and DBP < 80 mmHg. The exclusion criteria for both cohorts included terminal disease (e.g., cancer), diagnosis of dementia or other overt neurological diseases (e.g., Parkinson’s disease or stroke), history of brain trauma or injury, any health conditions making it difficult to perform the MRI (e.g., metal implant in the brain), and inability to understand the study protocol.

### Study Protocol

Each participant completed one screening visit and two study visits. On screening visit, the study personnel first introduced the study protocol to participants, and participants were then asked to answer three questions about the protocol (i.e., the study purpose, potential risks, and ability to withdraw from the study). Only when participants can answer all three questions correctly were they qualified for the ability to understand the study protocol. Next, each participant completed the questionnaires to assess the health and clinical characteristics, and a 1-minute walking test without any physical assistance. One study personnel monitored the walking test to ensure the safety of participants. Those who can complete the walking test without pause or stop were qualified for inclusion.

**FIGURE 1:**
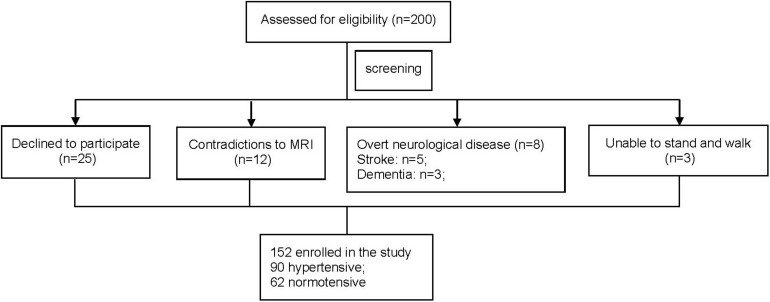
The flowchart of participant enrollment.

After the screening (Figure 1), a total of 152 eligible older adults were eligible for the following tests. Ninety participants had controlled hypertension, and the other 62 were normotensive. All the participants then completed two visits separated by 3 days. On the first visit, they completed one structural MRI of the brain to assess the grade of WMLs. On the second visit, they then completed the BP recordings followed by the tests of cognitive function and walking. All experimental methods and protocols were approved by the Institutional Review Board (IRB) of Shenzhen People’s Hospital and carried out in accordance with relevant guidelines. All the participants provided written consent for the participation in the study.

### MRI and Fazekas Scale

On the first visit, each participant completed one MRI scan for the whole-brain structure consisting of T1, T2, and FLAIR sequences on a 1.5-Tesla MR scanner. The specific parameters of the MRI scan were as follows: T1: repetition time (TR)/echo time (TE) = 250/2.48 ms, slice thickness = 5 mm, echo train length (ETL) = 1, matrix size = 320 mm × 320 mm; T2: TR/TE = 6,000/99 ms, slice thickness = 5 mm, ETL = 18, matrix size = 640 mm × 640 mm; and 3D-FLAIR: TR)/TE/inversion time (TI) = 9,000/2500/85 ms, slice thickness = 5 mm, echo train length (ETL) = 16, matrix size = 512 mm × 512 mm.

The severity grade of white matter lesions was then assessed separately by two neurologists using the Fazekas scale (Fazekas et al., 2002; Inzitari et al., 2009) based upon the brain structural MRI data of each participant. Both neurologists were blinded to the demographic and clinical information (e.g., hypertensive status) of all the participants. The Fazekas scale divided the white matter into periventricular and deep white matter, and each region is scaled at four grades ranging from 0 to 3 based upon the confluence and size of lesions. If different scales on one brain MRI were given by these two neurologists, a third neurologist was invited, and the scale was then discussed and determined by all three neurologists. The scale of the whole white matter (i.e., the sum of WMLs in periventricular and deep white matter) ranging from 0 to 6 was used in the following analysis. Greater Fazekas scale reflected higher WML grade of the brain.

### BP Recordings

On the second visit, each participant completed a BP assessment when sitting in a quiet assessment room with only one study staff. During the BP assessment, the participant was instructed to not talk and to keep motionless (e.g., not moving the arms). The objects which may interfere with the testing, such as the mobile phone, were stored in a box outside the room. The noninvasive finger blood pressure measurement was completed, which has been validated to brachial artery pressure (Guelen et al., 2008). Specifically, the continuous blood pressure series, including the systolic (SBP) and diastolic (DBP) BP, was recorded using the Finometer PRO system (Finapres Medical Systems B.V., Netherlands) at the middle finger of the left hand in supine position. The beat-to-beat blood pressure and inter-beat interval were recorded continuously for at least 10 min (10–15 min) with a sampling frequency of 100 Hz. The BeatScope software package (Finapres Medical Systems B.V., Netherlands) was then used to calculate the values of each beat. All the BP recordings thus consisted of at least 700 continuous beats of BP. The outliers in the BP series of which the value were greater or lower than the mean ± two standard deviation (SD) of the series which were interpolated by the mean. The preprocessed BP series with the length of 700 points were then used to obtain the complexity metric of BP.

### Multiscale Entropy

The *complexity* of beat-to-beat BP series was quantified using multiscale entropy (MSE), a technique that has been widely used to quantify the multiscale dynamics in the fluctuation of neurophysiological series (Costa et al., 2002, 2005). In MSE, the time series was first “coarse-grained” for different scales; that is, the original series was divided into nonoverlapping windows of length equaling a scale factor, τ. Here we used scales ranging from one to five sampling points. Thus, in the coarse-graining process, the series at Scale 1 was the raw time series consisting of 700 data points, which at Scale 2 was constructed by averaging every two nonoverlapped points, consisting of 350 points (i.e., 700 points/2), and the time series at the largest scale (i.e., Scale 5) had 140 data points (i.e., 700 points/5), which met the requirement for obtaining reliable estimates of sample entropy (Costa et al., 2005; Zhou et al., 2017). The sample entropies of each “coarse-grained” series were then calculated, by using the negative natural logarithm of the conditional probability that a time series, having repeated itself within a tolerance *r* for *m* points (pattern length), will also repeat itself for m + 1 points without self-matches. The sample entropy of each coarse-grained series in this study was computed by choosing *m* = 2 and *r* = 15%, which was suggested by previous studies (Costa et al., 2005). Finally, the BP complexity was defined as the averaged entropy across five scales. Lower MSE reflects lower complexity.

Additionally, the mean level of SBP and DBP was used in the following analyses.

### Walking Assessment

Following the BP assessment, each participant completed the walking assessment, consisting of two 10-meter trials (Peters et al., 2013; Amatachaya et al., 2014; Chan and Pin, 2019) under each of the following conditions: walking quietly (i.e., single-task) and walking while performing a verbalized serial subtraction of three test (i.e., dual-task). They were asked to walk at their preferred speed along a 10-meter straight hallway. The walking speed was measured using the Mobility Lab^TM^ system (APDM, Portland, and OR), a commercialized wearable system, consisting of six motion sensors to capture the kinematic data related to walking (Mancini et al., 2011). In dual-task trials, a three-digit random number was given by the study staff at the beginning of each trial, and the participant was asked to perform the serial subtraction throughout the trial. The walking speed of each trial was then measured, and the walking speed averaged across two trials within each condition was used in the following analyses.

### Mini-Mental State Examination

The Chinese version of MMSE (Folstein et al., 1983; Katzman et al., 1988) was used to assess the general cognitive function of each participant. The total score of MMSE was used in the analysis, and a lower score reflected poorer cognitive function.

### Statistical Analysis

Data distribution and missing values were checked for all variables. Analysis of variance (ANOVA) models were used to examine the association between hypertension and walking speed in single- and dual-task conditions. The dependent variable was single- or dual-task walking speed in separate models, and the factor was group (i.e., hypertensive and normotensive). Similar models were also used to examine the difference in other functional outcomes (i.e., Fazekas scale, MMSE score, and BP complexity) between hypertensive and normotensive cohorts. Age was used as the covariate in these models.

Linear regression models were then used to examine the relationship of walking speeds to complexity of SBP and DBP, MMSE score, and Fazekas scale across all the participants. Age and group were included as covariates in these regression models, because studies have shown that older age is a major contributor to slowed walking speed, poor MMSE score, and increased WMLs (Piccinin et al., 2013; Habes et al., 2016; Takayanagi et al., 2019), and the status of BP (i.e., group: hypertensive and normotensive) also had influences on these outcomes. The relationship between mean level of SBP and DBP and walking speeds was also examined using similar models.

Then, the structural equation modeling (SEM) with robust maximum likelihood estimation was used to examine the direct and indirect effects of hypertension on single- and dual-task walking speeds and to examine the possible factors (i.e., BP complexity, Fazekas scales, MMSE) that may mediate the effects of hypertension on walking speed. BP complexity, Fazekas scales, and MMSE score were added in the multivariate models consecutively and simultaneously. The coefficients of each individual path were tested for statistical significance, and direct, indirect, and total effects of each variable on walking speed were assessed. The outcome variables (i.e., single- and dual-task walking speed) were examined in separate models. The SEM analyses were completed with and without adjusting for age. For each SEM model, the *p*-value for the χ^2^ statistic, root mean square error of approximation (RMSEA), comparative fit index (CFI), and Tucker–Lewis index (TLI) were used as model indices to assess model fits. A *p*-value > 0.05 for the χ^2^ statistic, RMSEA < 0.06, CFI, and TLI ≥ 0.95 were considered good model fits (Schreiber et al., 2006).

The significance level for all these models was set at *p* < 0.05. Statistical analyses were performed with JMP Pro. 14 software (SAS Institute, Cary NC) and Mplus 8.4 (Muthén and Muthén, Los Angeles, CA, United States).

## Results

All the participants completed the tests of this study, and their data were included in the analyses. Table 1 shows the demographics and clinical and functional outcomes of the entire cohort and of the two groups (i.e., hypertensive, and normotensive cohorts) separately. No significant differences in age, sex, and BMI were observed between the two groups (*p* > 0.18).

**TABLE 1:** Demographics and clinical and functional information of the participants.

**Mean ± S.D.**		**Total cohort (*n* = 152)**	**Hypertension cohort (*n* = 90)**	**Normotensive cohort (*n* = 62)**	***p* value**
Age (years)		71.88.9	738.8	719	0.18
Sex (female)		*n* = 69	*n* = 40	*n* = 30	0.76
BMI		23.83.5	24.23.6	23.13.2	0.22
MMSE		26.14.6	25.44.4	27.42.8	0.006
Fazekas scale	Total	2.71.4	3.11.3	2.21.4	0.002
	Periventricular	1.40.8	1.50.7	1.10.8	0.03
	Deep	1.30.8	1.50.8	1.10.8	0.01
Main BP level	SBP	13718.5	144.315.2	125.813.2	<0.0001
	DBP	78.68.9	80.29.4	75.27.3	0.001
Walking speed	Single-task	0.850.27	0.790.27	0.920.23	0.002
	Dual-task	0.770.24	0.720.26	0.830.21	0.003
Complexity	SBP	1.450.28	1.40.24	1.510.26	0.004
	DBP	1.370.32	1.270.33	1.450.29	0.001

### The Effects of Hypertension on the Clinical and Functional Characteristics

Two-way ANOVA models demonstrated significant effects of hypertension on clinical and functional characteristics (Table 1). Specifically, as compared to normotensive, the hypertensive group had a significantly lower MMSE score (*F* = 7.3, *p* = 0.006, i.e., poorer cognitive function), higher total Fazekas score (*F* = 8.4, *p* = 0.004), and scores within periventricular (*F* = 6.5, *p* = 0.03) and deep (*F* = 5.2, *p* = 0.01) white matter (i.e., worse WMLs), slower walking speeds in both single- (*F* = 9.7, *p* = 0.002) and dual-task conditions (*F* = 8.3, 0.004), and lower complexity of SBP (*F* = 7.9, *p* = 0.004) and DBP (*F* = 11.3, 0.001). All the significance was independent from age.

### The Relationship of Walking Speeds to BP Complexity, WML Grade, and Cognitive Function

**FIGURE 2:**
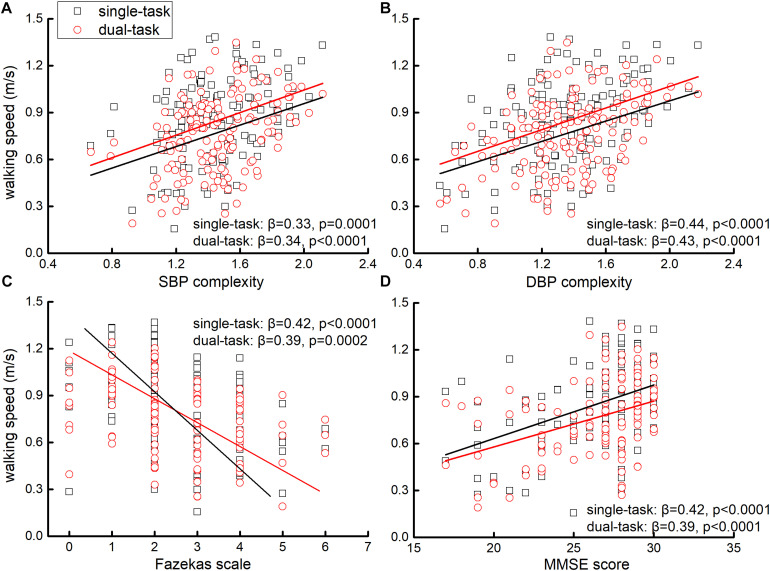
The association between SBP **(A)** and DBP complexity **(B)**, Fazekas scale **(C)**, MMSE score **(D)**, and walking speeds in single- and dual-task conditions. Liner regression analyses showed that those with lower complexity of SBP and/or DBP, greater Fazekas scale (i.e., greater grade of WMLs), and/or lower MMSE score (i.e., poorer cognitive function) performed slower single- and dual-task walking speeds.

Linear regression analyses adjusted for age and group showed that across all participants, the complexity of SBP and DBP was significantly associated with walking speed in single- and dual-task conditions. Specifically, participants with lower complexity of SBP and/or DBP had slower walking speed in single- and/or dual-task conditions (β > 0.33, *p* < 0.0001, Figures 1B and 2A). Within each group (i.e., within hypertensive or normotensive), such significant association between BP complexity and walking speed was also observed (β > 0.31, *p* < 0.003). A similar association was also observed between Fazekas scale, MMSE score, and walking speed in single- and dual-task conditions, that is, participants with greater Fazekas scale (β < −0.39, *p* < 0.001 Figure 2C) and/or lower MMSE score (β < −0.39, *p* < 0.001, Figure 2D) walked slower in single- and/or dual-task conditions. No such significant association was observed between mean level of complexity of SBP and DBP and walking speed (β < 0.15, *p* > 0.49). Additionally, we also found that lower BP complexity was associated with greater Fazekas scale (β > 0.45, *p* < 0.001).

### The Interrelationships Between Hypertension, BP Complexity, WML Grade, and Cognitive Function, and Walking Speeds as Assessed by Structural Equation Modeling

**FIGURE 3:**
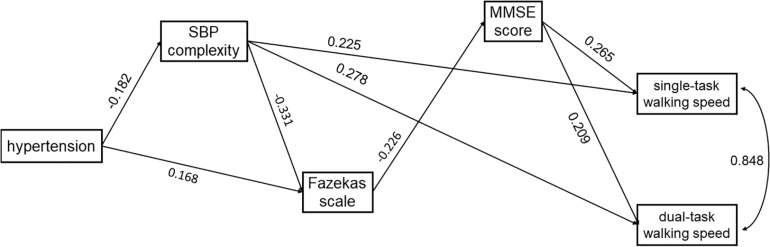
The SEM model assessing the interrelationships between hypertension, SBP complexity, Fazekas scale, MMSE score, and walking speed in single- and dual-task conditions. The model was adjusted for age, and only the significant paths are presented.

**FIGURE 4:**
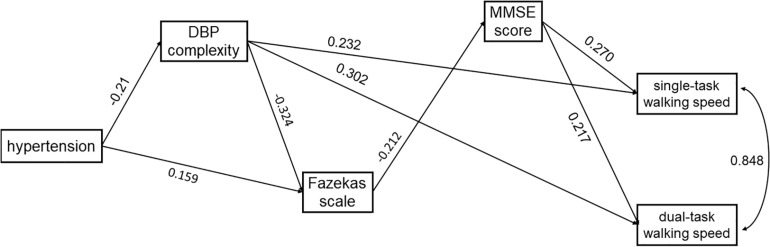
The SEM model assessing the interrelationships between hypertension, DBP complexity, Fazekas scale, MMSE score, and walking speed in single- and dual-task conditions. The model was adjusted for age, and only the significant paths are presented.

Two age-adjusted SEM models were performed for SBP (i.e., Model 1) and DBP (i.e., Model 2) complexity separately. Tables 2 and 3 and Figures 3 and 4 show the detailed results of the two models. Both models showed significant total effects of hypertension on walking speed in single- (Model 1: −0.138, *p* = 0.013; Model 2: −0.139, *p* = 0.012) and dual-task (Model 1: −0.153, *p* = 0.010; Model 2: −0.155, *p* = 0.009) conditions, but no significant direct effects or total indirect effects of hypertension on walking speeds were observed (*p* > 0.05). The complexity of SBP (Model 1) or DBP (Model 2), score of Fazekas scale, and MMSE score mediated the association between hypertension and walking speed, accounting for 26∼39% of the total effects of hypertension on single- (Model 1: 26%; Model 2: 36%) and dual- (Model 1: 27%; Model 2: 39%) task walking speed.

**TABLE 2:** Total, direct, and indirect standardized effects of hypertension on walking speeds as assessed by SEM model including the complexity of SBP (Model 1).

**Paths**	**Estimate**	**SE**	**95% CI**		***p*-value**
			**Lower**	**Upper**	
**Hypertension → single-task walking speed**					
Total effects	−0.138*	0.056	−0.248	−0.029	0.013
Direct effects	−0.103	0.057	−0.215	0.010	0.074
Total indirect effects	−0.036	0.025	−0.085	0.013	0.154
Hypertension → SBP → single-task walking speed	−0.041	0.022	−0.084	0.002	0.064
Hypertension → Fazekas → single-task walking speed	0.014	0.016	−0.018	0.046	0.394
Hypertension → SBP → Fazekas → single-task walking speed	0.005	0.006	−0.006	0.016	0.371
Hypertension → Fazekas → MMSE → single-task walking speed	−0.010	0.007	−0.024	0.004	0.153
Hypertension → SBP → Fazekas → MMSE → single-task walking speed	−0.004	0.003	−0.009	0.001	0.150
**SBP → single-task walking speed**					
Total effects	0.217***	0.052	0.116	0.319	<0.001
Direct effects	0.225***	0.059	0.110	0.340	<0.001
Total indirect effects	−0.008	0.027	−0.060	0.045	0.779
SBP → Fazekas → single-task walking speed	−0.027	0.027	−0.081	0.026	0.318
SBP → Fazekas → MMSE → single-task walking speed	0.020*	0.010	0.001	0.038	0.037
**Fazekas → single-task walking speed**					
Total effects	0.023	0.081	−0.135	0.180	0.778
Direct effects	0.083	0.080	−0.075	0.240	0.303
Total indirect effects	−0.060*	0.027	−0.112	−0.008	0.024
Fazekas → MMSE → single-task walking speed	−0.060*	0.027	−0.112	−0.008	0.024
**Hypertension → dual-task walking speed**					
Total effects	−0.153*	0.059	−0.269	−0.037	0.010
Direct effects	−0.112	0.061	−0.231	0.007	0.065
Total indirect effects	−0.041	0.029	−0.099	0.016	0.160
Hypertension → SBP → dual-task walking speed	−0.051	0.027	−0.103	0.001	0.057
Hypertension → Fazekas → dual-task walking speed	0.015	0.018	−0.022	0.051	0.426
Hypertension → SBP → Fazekas → dual-task walking speed	0.005	0.404	−0.007	0.018	0.404
Hypertension → Fazekas → MMSE → dual-task walking speed	−0.008	0.006	−0.019	0.003	0.163
Hypertension → SBP → Fazekas → MMSE → dual-task walking speed	−0.003	0.002	−0.007	0.001	0.167
**SBP → dual-walking speed**					
Total effects	0.265***	0.058	0.152	0.378	<0.001
Direct effects	0.278***	0.068	0.145	0.412	<0.001
Total indirect effects	−0.013	0.031	−0.075	0.048	0.668
SBP → Fazekas → dual-walking speed	−0.029	0.032	−0.091	0.033	0.360
SBP → Fazekas → MMSE → dual-walking speed	0.016	0.008	0.000	0.032	0.056
Fazekas → dual-task walking speed					
Total effects	0.041	0.094	−0.143	0.224	0.665
Direct effects	0.088	0.093	−0.095	0.270	0.346
Total indirect effects	−0.047*	0.023	−0.092	−0.002	0.039
Fazekas → MMSE → dual-task walking speed	−0.047*	0.023	−0.092	−0.002	0.039

*p < 0.05; ***p < 0.001.

**TABLE 3:** Total, direct, and indirect standardized effects of hypertension on walking speeds as assessed by SEM model including the complexity of DBP (Model 2).

**Paths**	**Estimate**	**SE**	**95% CI**		***p*-value**
			**Lower**	**Upper**	
**Hypertension → single-task walking speed**					
Total effects	−0.139*	0.056	−0.248	−0.030	0.012
Direct effects	−0.089	0.059	−0.204	0.026	0.128
Total indirect effects	−0.050	0.026	−0.101	0.002	0.059
Hypertension → DBP → single-task walking speed	−0.049*	0.023	−0.094	−0.004	0.034
Hypertension → Fazekas → single-task walking speed	0.008	0.014	−0.018	0.035	0.537
Hypertension → DBP → Fazekas → single-task walking speed	0.004	0.005	−0.007	0.014	0.499
Hypertension → Fazekas → MMSE → single-task walking speed	−0.009	0.007	−0.023	0.005	0.190
Hypertension → DBP → Fazekas → MMSE → single-task walking speed	−0.004	0.002	−0.008	0.001	0.090
**DBP → single-task walking speed**					
Total effects	0.234***	0.062	0.113	0.354	<0.001
Direct effects	0.232***	0.064	0.106	0.359	<0.001
Total indirect effects	0.001	0.025	−0.047	0.050	0.952
DBP → Fazekas → single-task walking speed	−0.017	0.025	−0.065	0.031	0.487
DBP → Fazekas → MMSE → single-task walking speed	0.019*	0.009	0.001	0.036	0.033
**Fazekas → single-task walking speed**					
Total effects	−0.005	0.076	−0.153	0.144	0.952
Direct effects	0.052	0.075	−0.094	0.199	0.484
Total indirect effects	−0.057*	0.026	−0.108	−0.006	0.029
Fazekas → MMSE → single-task walking speed	−0.057*	0.026	−0.108	−0.006	0.029
**Hypertension → dual-task walking speed**					
Total effects	−0.155**	0.059	−0.270	−0.039	0.009
Direct effects	−0.094	0.062	−0.215	0.026	0.126
Total indirect effects	−0.060	0.031	−0.122	0.001	0.054
Hypertension → DBP → dual-task walking speed	−0.063*	0.028	−0.119	−0.008	0.025
Hypertension → Fazekas → dual task walking speed	0.010	0.015	−0.021	0.040	0.538
Hypertension → DBP → Fazekas → dual-task walking speed	0.004	0.006	−0.008	0.016	0.501
Hypertension → Fazekas → MMSE → dual-task walking speed	−0.007	0.006	−0.018	0.004	0.198
Hypertension → DBP → Fazekas → MMSE → dual-task walking speed	−0.003	0.002	−0.007	0.001	0.106
**DBP → dual-task walking speed**					
Total effects	0.298***	0.063	0.174	0.422	<0.001
Direct effects	0.302***	0.068	0.168	0.415	<0.001
Total indirect effects	−0.005	0.028	−0.060	0.051	0.873
DBP → Fazekas → dual-walking speed	−0.019	0.028	−0.075	0.036	0.491
DBP → Fazekas → MMSE → dual-walking speed	0.015*	0.008	0.000	0.030	0.048
**Fazekas → dual-task walking speed**					
Total effects	0.014	0.087	−0.156	0.184	0.873
Direct effects	0.060	0.085	−0.108	0.227	0.485
Total indirect effects	−0.046*	0.023	−0.090	−0.002	0.042
Fazekas → MMSE → dual-task walking speed	−0.046*	0.023	−0.090	−0.002	0.042

*p < 0.05‘’ **p < 0.01; ***p < .001.

In both models, hypertension was directly associated with lower complexity of SBP (Model 1: standardized coefficient = −0.182, *p* = 0.017) or DBP (Model 2: standardized coefficient = −0.210, *p* = 0.003). Hypertension was also directly (Model 1: standardized coefficient = 0.168, *p* = 0.031; Model 2: standardized coefficient = 0.159, *p* = 0.047) or indirectly (Model 1: standardized coefficient = 0.060, *p* = 0.054; Model 2: standardized coefficient = 0.068, *p* = 0.023) associated with the score of the Fazekas scale *via* complexity of SBP (Figure 3) or DBP (Figure 4). Walking speed in single- and dual-task conditions was highly correlated with each other (standardized coefficient = 0.848, *p* < 0.001). The score of the Fazekas scale was in turn indirectly associated with single- and dual-task walking speed *via* MMSE score (standardized coefficient < −0.047, *p* < 0.042). The complexity of SBP and DBP was each directly (standardized coefficient = 0.225∼0.302 *p* < 0.001) and indirectly (standardized coefficient = 0.015∼0.033, *p* = 0.037∼*p* = 0.056) associated with single- and dual-task walking speed *via* the score of the Fazekas scale and MMSE score. Significant total effects of BP complexity on walking speed were observed in single- (Model 1: 0.217, *p* < 0.001; Model 2: 0.234, *p* < 0.001) and dual- (Model 1: 0.265, *p* < 0.001; Model 2: 0.298, *p* < 0.001) task conditions. Additionally, in Model 2, hypertension was indirectly associated with single- (standardized coefficient = −0.049, *p* = 0.034) and dual-task (standardized coefficient = −0.063, *p* = 0.025) walking speed *via* DBP complexity.

All these variables from two models explained 49% (R-squared = 0.489 for SBP complexity and 0.492 for DBP complexity, *p* < 0.001) of variation of single-task walking speed, and 40% (SBP complexity: R-squared = 0.404, *p* < 0.001) and 42% (DBP complexity: R-squared = 0.416, *p* < 0.001) of variation of dual-task walking speed. The model exhibited a perfect model fit with χ^2^ (*df* = 2) = 0.088∼0.828, *p* = 0.661∼0.957, RMSEA = 0.00, CFI = 1.00 and TLI = 1.00.

## Discussion

The altered regulation of BP in hypertension has been linked to multiple complications in older adults. Similar to previous studies (Dumurgier et al., 2010; Rosano et al., 2011), we here observed elevated grade of WMLs, diminished cognitive function, and decreased walking speed in the hypertensive cohort as compared to normotensives. The WMLs and cognitive function were each associated with walking speeds in single- and dual-task conditions. To further explore the potential mechanism through which hypertension affects walking speed, we characterized the multiscale dynamics of the continuous beat-to-beat SBP and DBP fluctuation using complexity and examined interrelationships between hypertension, BP complexity, grade of WMLs, cognitive function, and walking speed in single- and dual-task conditions. The results demonstrated that (1) the BP complexity is associated with walking speed, that is, older adults with lower complexity of SBP and/or DBP walked more slowly, and (2) such BP complexity is a strong and independent factor contributing to walking speed and mediates the relationship between hypertension and walking speed in older adults. Thus, the complexity of continuous beat-to-beat BP fluctuation may provide unique insight into the vascular mechanisms pertaining to the regulation of walking and may potentially serve as a target in future rehabilitative strategies for walking performance in older adults.

The theory of complexity in aging proposes that the regulation of a given physiologic system (e.g., cardiovascular system) consists of multiple structural and functional elements, and these elements are communicating with each other over multiple scales of time and space (Manor and Lipsitz, 2013). Blood pressure, for example, is determined by the cardiac output and the systemic vascular resistance and maintained by multiple underlying neural and hormonal feedback mechanisms, including resistance vessels, baroreceptors, and sympathetic and parasympathetic nervous systems (Dauphinot et al., 2013). Aging and age-related conditions (e.g., hypertension) often diminish the quantity and/or quality of those elements as well as their interaction within the system, leading to the loss of the system’s functionality adapting to stress (Manor et al., 2010). Studies (Costa et al., 2002; Zhou et al., 2020b) have linked the diminished degree of the physiologic complexity within the spontaneous out-fluctuations of the system to the age-related loss of the system’s functionality. For example, Costa et al. (2002) found that compared to the healthy cohort, the complexity of heartbeat time series (as quantified using MSE) in people with chronic heart failure or with atrial fibrillation was significantly lower. In this study, we quantified the complexity of best-to-beat BP fluctuation using MSE and linked the physiologic complexity of the cardiovascular system to slowed walking speed in older adults. Future longitudinal studies are worth characterizing the elements within the vascular system (e.g., stiffness of artery) and exploring how the changes in these elements are associated with the changes in BP complexity.

The results of the SEM models showed a significant total effect of hypertension on walking speed in both single- and dual-task conditions, but such effect is mediated by BP complexity, WMLs, and cognitive function. Specifically, a conceptual framework may reveal the underlying mechanism of that effect; that is, hypertension may disrupt the regulation of blood pressure by altering elements in the cardiovascular system (e.g., increased vessel stiffness), which can be manifest as the decrease in BP complexity. The disrupted cardiovascular regulation then influences the regulation of the cerebral vascular system, leading to increased WML grade. The WMLs limit the interaction and communication between different brain regions and diminish cognitive resources pertaining to control of walking, leading to slowed walking speed in hypertensive older adults. Among these interrelationships, it is also observed that the BP complexity is directly associated with the walking speed, and such relationship is independent from other metrics included in these models (i.e., Fazekas scale, MMSE score, age). Moreover, only BP complexity (not WMLs or MMSE) significantly mediates the relationship between hypertension and walking speed, accounting for up to 41% of such relationship. This evidence further suggests that, in addition to the “hypertension-complexity-WMLs-MMSE-walking speed” mechanism, other mechanisms of hypertension-related effect on walking may exist and are warranted to be explored in future studies.

It should be noted that the BP fluctuation was recorded in a short time length (∼15 min) at resting state and the BP complexity was measured based upon this “resting-state” BP fluctuation in this study. Previous studies, on the other hand, showed that the fluctuation in the ambulatory BP across hours or days was associated with important health characteristics (e.g., WMLs, cognitive function, etc.) in older adults (Sierra, 2011; Cho et al., 2018; Lodhi et al., 2019). Cho et al. (2018), for example, demonstrated that in older adults with one or more cardiovascular risk factors, higher variability of 24-h ambulatory BP was associated with cognitive impairment, indicating that the characterization of the continuous *ambulatory* BP patterns may provide critical knowledge to the regulation of vascular systems and may help optimize BP management in older adults. However, the multiscale dynamics of the ambulatory BP remain unknown, which are worthwhile to be explored in future studies. Additionally, in this study, the calculation of MSE focused on a relatively short range of the scales (i.e., scale 1 to 5) due to the limitation of the BP recording length. Therefore, the characterization of the complexity of ambulatory BP fluctuations over a longer range of temporal scales may provide important knowledge of the vascular regulation beyond the scales this study explored.

The observed difference in the functional outcomes between hypertensive and normotensive cohorts in this study may indicate clinically meaningful differences as induced by hypertension. Specifically, as compared to the normotensive group, the hypertensive group had a significantly lower MMSE score with a mean difference of two scores. This meets the minimal clinically important difference (MCID) of the MMSE score as suggested by Andrews et al. (2019), indicating a significant impact of hypertension on cognitive function in older adults. Previous studies also showed the MCID of walking speed that is associated with the survival rate and the risk of dementia in older adults (Studenski et al., 2011; Montero-Odasso et al., 2017). Based upon the observations from Montero-Odasso et al. (2017), for example, older adults with dual-task walking speed slower than 0.81 m/s had the highest risk of dementia. The mean walking speed in the hypertensive group in our study was 0.72 m/s, indicating that this hypertensive cohort is of great risk of dementia in the near future. Therefore, it is critical to optimize the rehabilitative strategies that can induce clinically meaningful improvement of cognitive-motor function in older adults. The observations here suggest that such rehabilitative strategies may be optimized by targeting the complexity of BP fluctuation. Multiple studies have shown that the loss of physiologic complexity is not an obligatory consequence of aging or age-related diseases but can be restored with appropriate interventions (Millar et al., 2013; Ma et al., 2019; Zhou et al., 2020a). For example, Millar et al. (2013) showed that in a hypertensive cohort, 8-week isometric hand-grip training can help control the BP by lowering the BP level and increase the complexity of heart rate fluctuation as quantified using detrended fluctuation analysis. Future studies are thus worthwhile to explore the effects of interventions (e.g., anti-hypertension medication) on BP complexity and to determine if the increase in the BP complexity is associated with clinically meaningful improvements in walking and cognitive function in older adults.

It should be noted that this is a cross-sectional study with a relatively small sample size, so that no causal relationship between these functions can be established, which is warranted to be examined in future longitudinal studies of a larger sample size. All the participants in the hypertensive group have taken the anti-hypertension medication for certain time, and the potential effects of the medication on the BP complexity and other outcomes were not explored due to the lack of the medication information. Future studies are needed to assess the effects of medication (e.g., length of taking medication) on the observed interrelationships. The WMLs were assessed using the Fazekas scale, a clinical rating scale, and only the general cognitive function was measured using MMSE. Sophisticated measurement of WMLs [e.g., the integrity of white matter tracks as quantified using diffusion weighted MRI (Jones et al., 2013)] and specific assessments to different cognitive domains (e.g., working memory or processing speed) are worthwhile to be measured in future studies. Additionally, we only assessed the walking speed. Previous studies showed that the speed and variability of gait when walking (i.e., stride time variability) were associated with different functional brain networks in older adults (Lo et al., 2017). It is thus necessary in future studies to explore how hypertension affects other aspects of walking performance, such as gait variability, which may help provide insight into the potential mechanisms through which hypertension affects the regulation of walking in older adults.

Nevertheless, this study provides the novel proof-of-concept of a potential underlying mechanism through which hypertension may affect walking speed in both single- and dual-task conditions in older adults. The physiologic complexity of continuous beat-to-beat BP series is a contributor to such relationship, which may serve as a novel marker for the management of hypertensive older adult population.

## Data Availability Statement

The raw data supporting the conclusions of this article will be made available by the authors, without undue reservation.

## Ethics Statement

The studies involving human participants were reviewed and approved by Institutional Review Board (IRB) of Shenzhen People’s Hospital. The patients/participants provided their written informed consent to participate in this study.

## Author Contributions

XJ and JZ designed the study. XJ, DP, HZ, WD, WF, NQ, and RC collected the data. XJ, YC, YZ, and JZ analyzed the data and performed statistical analyses. XJ, YC, and JZ interpreted the results and drafted the manuscript. All authors contributed to and approved the final version.

## Conflict of Interest

The authors declare that the research was conducted in the absence of any commercial or financial relationships that could be construed as a potential conflict of interest.
